# Neuropsychology in the Times of COVID-19. The Role of the Psychologist in Taking Charge of Patients With Alterations of Cognitive Functions

**DOI:** 10.3389/fneur.2020.573207

**Published:** 2020-10-15

**Authors:** Matteo Sozzi, Lorella Algeri, Matteo Corsano, Davide Crivelli, Maria Angela Daga, Francesca Fumagalli, Paola Gemignani, Maria Concetta Granieri, Maria Grazia Inzaghi, Francesca Pala, Simone Turati, Michela Balconi

**Affiliations:** ^1^Department of Neuroscience, Neurology Unit, ASST “A. Manzoni,” Lecco, Italy; ^2^Board of Società degli Psicologi dell'Area Neuropsicologica (SPAN), Civate, Italy; ^3^Psychology Unit, ASST “Papa Giovanni XXIII,” Bergamo, Italy; ^4^Center for Rehabilitation, Consorzio San Stef. Ar. Abruzzo, Pescara, Italy; ^5^Research Unit in Affective and Social Neuroscience, Catholic University of the Sacred Heart, Milan, Italy; ^6^Department of Psychology, Catholic University of the Sacred Heart, Milan, Italy; ^7^Department of Neurorehabilitation Sciences, Casa di Cura Privata del Policlinico, Milan, Italy; ^8^Levante Ligure Rehabilitation Center, Fondazione Don C. Gnocchi Onlus, La Spezia, Italy; ^9^Associazione “J.F. Kennedy” - Centro di Riabilitazione Neuropsicomotoria ONLUS, Acireale, Italy; ^10^Laboratory for Neuropsychology, Istituto Clinico Quarenghi, San Pellegrino Terme, Italy

**Keywords:** neuropsychology, COVID-19, healthcare, cognitive impairment, neuropsychological assessment, neurorehabilitation, neuro-COVID

## Introduction: State of the Art and the Role of the Psychologist

From the very beginning of this severe pandemic, the intervention of psychologists in managing and containing the spreading infection has been essential. Now that, in some respects, the emergency for protection of human lives is receding, another opportunity for psychologists' intervention has emerged: the neuropsychological field.

Recent scientific publications highlight the cognitive sequelae in neuro-COVID syndromes: tropism of this type of virus for central nervous system and prolonged periods of hypoxia due to severe desaturation represent highly significant factors in determining dysfunctional alterations of cognitive functioning. Indeed, it is widely known that protracted hypoxemic episodes cause cognitive impairments. For instance, Hopkins et al. (1) have pointed out that about 50% of patients with acute respiratory distress disorder (ARDS) shows cognitive alterations up to 2 years after the acute event. Consistently, the authors recommend carrying out an assessment of memory and executive functions in order to foster long-term monitoring processes.

Concerning the etiopathogenetic nature of COVID-19, the virus' distinctive features favor its access up to the blood–brain barrier via retrograde axonal transport along cranial nerves—in particular, the olfactory nerve, which explains one of the most frequently reported symptoms. The alteration of the blood–brain barrier determines the onset of neurological conditions known as necrotizing acute encephalopathies (2–4). Moreover, patterns of brain lesions have been documented, with hemorrhagic effusions at well-defined hemorrhagic rims around the thalamus, in subregions of the medial temporal lobes, and in subinsular regions (4).

Clinical features of COVID-19 are to be considered according to the severity of their manifestation: the clinical picture is defined as *mild* or *asymptomatic* when the disease occurs in the absence of dyspnea or desaturation; *moderate* when the O_2_ saturation is between 94 and 98% and there are signs of pneumonia on radiological examination; *severe* in case of O_2_ saturation below 93%, increased of respiratory rate, interstitial pneumonia, and need to add O_2_ to the natural respiratory process; and *critical* when, in addition, mechanical ventilation is required.

Generally, patients with mild COVID-19 symptomatology recover with no need of specific interventions, whereas in moderate, severe, and critical forms, several systems, first and foremost the CNS, suffer from implications of the infection. Persistent desaturation levels are associated with worsening dyspnea, which, in turn, has serious repercussions on brain metabolism (5). In their work, Carda et al. (5) also report the clinical experience with a sample of Italian COVID-19 patients, which have presented cognitive alterations such as memory disorders, deficits of executive functions and, among older subjects with severe forms, confusion [see also (6, 7)]. These impairments are due to the effect of the viral infection on the central nervous system, as previously mentioned, and to long periods of hypoxygenation and brain injuries, as clearly reported by Girardini et al. (8). Likewise, Li et al. (9) report, in a study conducted on a sample of 211 patients, that patients with severe infection develop cerebrovascular impairment syndromes. In a systematic review, it was confirmed that one out of four patients with ARDS consequent to COVID-19 infection develops neuropsychological symptoms as a manifestation of CNS involvement (10). Hence, it is strongly recommended that early identification and care of cognitive deficits should be performed. Neurological deficits involve, among others, severe changes in the state of consciousness and consequent alterations of cognitive functions (7).

**Figure 1:**
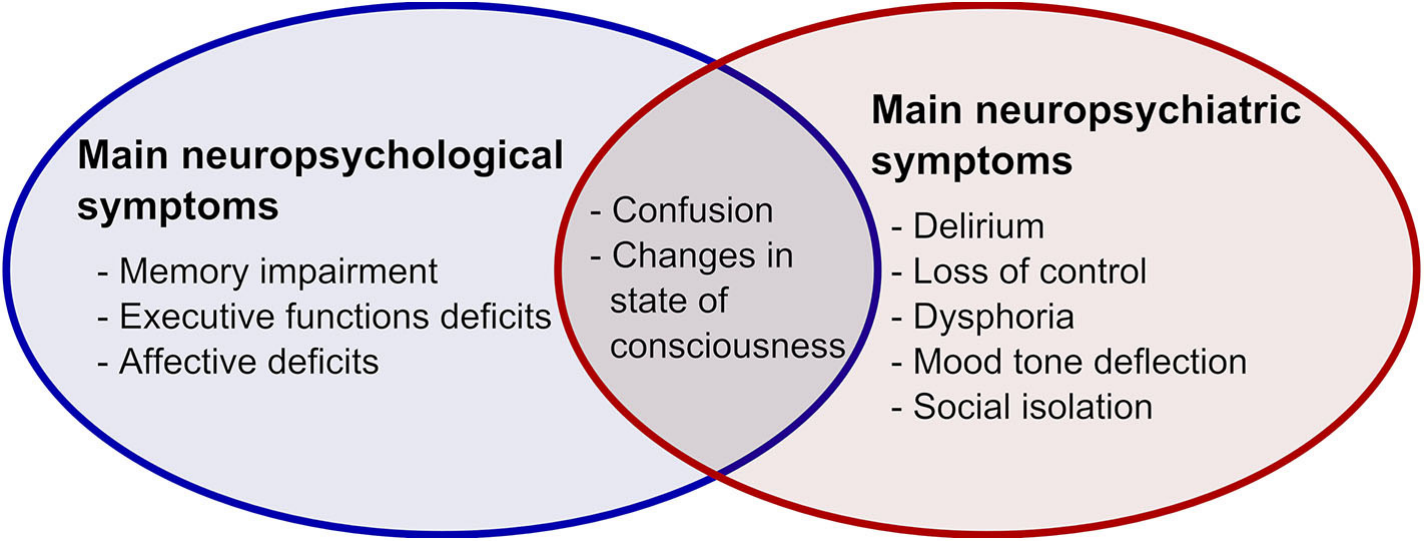
Main neuropsychological and neuropsychiatric symptoms in COVID-19 patients.

What has been so far highlighted by scientific evidence seems sufficiently convincing to demonstrate that this type of lung disease can remarkably affect the CNS and that cognitive, emotional, and behavioral functions may be severely compromised (11, 12). Indeed, behavioral alterations compatible with delirium and loss of control have also been found as a consequence of hypoxemia and cerebral lesions. On the other hand, thymic alterations, such as dysphoria and mood tone deflection, are frequently associated with isolation and sudden loss of meaningful social contacts with significant family members. Figure 1 reports main neuropsychological and neuropsychiatric symptoms in COVID-19 patients.

## Proposal for the Current Situation: Neuropsychological Assessment

Field experience shared by colleagues working in these areas points out that, in these days, neuropsychologists are facing a constantly increasing number of requests for assessment and care of patients showing cognitive outcomes as a result of the new coronavirus infection.

It is reasonable to think that when all concerns about the potential risk for transmission of the contagion and isolation have ceased and people are to resume their usual working activities, the invalidating cognitive consequences will emerge with strength and the demand for neuropsychological intervention will further increase.

In our opinion, it is necessary to boost neuropsychological services and, consequently, the number of psychologists serving in the neuropsychology field, in order to cope with the increase in demand. Effective measures to be taken with this kind of patients involve—in addition to the dissemination of information concerning the high risk of cognitive repercussions—neuropsychological assessment, rehabilitation treatment, and the role of the psychologist/neuropsychologist in managing cognitive issues. Table 1 summarizes recommendations for neuropsychologists' practice.

**Table 1:** Summary of recommendation for neuropsychologists.

Assessment process	Online neuropsychological assessment vs. in presentia assessment	Benefit	Limitations	Recommendations
		- Simpler test administration when online - Observance of safety norms even in critical conditions	- Impossibility of making adequate clinical and qualitative observations of emotional–cognitive–behavioral alterations - Limitation in socio-relational factors	- A vis-à-vis approach is recommendable, unless urgent conditions imply that the evaluation cannot be postponed
	Tests administration	Measures	Test administration condition	Recommendations
		- Global functionality, cognitive flexibility, problem-solving skills, working memory, and praxis abilities, as well as mnesic, learning, and attention skills - Assessment of patient's residual abilities - Detection and quantification of possible affective alteration	- Compatibility with patient's clinical conditions - Retention of minimal attention levels	- Be sure that COVID-related clinical condition (i.e., SARS, neurological and motor deficits) is emended - Adequate psychometric properties of selected tests
Rehabilitative intervention	Online neuropsychological intervention and in presentia rehabilitation: features	In presentia rehabilitation	Online intervention	Recommendations
		- Calibrating the commitment required from the patient for an effective rehabilitation - Support deficit recovery or maintenance over time	- Planning of teletherapy intervention - Remote administration of exercises - Modulation of gradients of difficulty based on the actual level of impairment - Constant monitoring by the neuropsychological expert	- Both solutions are strongly recommended, especially with effective and planned exercises aimed at functional cognitive recovery - Choose between online and in presentia rehabilitation based on both sanitary global condition (quarantine, lockdown, etc.) and patient's/caregiver's needs and resources

Neuropsychological assessment, which cannot be considered as the mere administration of psychometric tests, provides a profile of residual abilities, emerging difficulties, and potential trend of cognitive decline, just as it occurs for other neurological diseases. The assessment procedures, then, provide relevant information to outline opportunities for intervention. The psychologist is called to take part into this clinical decision-making process based on specific neuropsychological expertise and competence. In the backdrop of this epidemic, the possibility to carry out an online neuropsychological assessment, by using telematic administration of tests, has also been brought to attention.

In a special note sent to the National Council of the Order of Psychologists on May 8, 2020 (13), we had the chance to express our opinions and some recommendation concerning remote neuropsychological assessment by referring to the scientific literature and by following the Guidelines of the American Psychological Association. Online assessment exhibits a number of limitations in the process of evaluating cognitive functions. In particular, remote procedures miss crucial steps because of the impossibility of making adequate qualitative clinical observations concerning patients' affective–cognitive–behavioral skills, as well as specific socio-relational dimensions. Having said that, while it has to be acknowledged that a few specific tools for remote assessment have already been presented in peer-reviewed journals, we argue that they should be used for such purpose if, and only if, the values of the normative sample can be properly applied to and compared with the patient. For these and other reasons—referring to which we suggest to read the abovementioned note—we conclude that a vis-à-vis approach is still recommendable, unless urgent conditions imply that the evaluation cannot be postponed.

The assessment of COVID-19 patients cannot be limited to the administration of screening batteries that provide scores mirroring the so-called “global functionality” or, at the very least, it cannot stop at such level of analysis because this would mean denying the patients the right and possibility to undergo a proper evaluation of the cognitive profile. According to the extant literature, the assessment should include the administration of tests that accurately evaluate cognitive flexibility, problem-solving skills, and working memory, as well as mnesic, learning, and attention skills. Nonetheless, the assessment session should also be compatible with patient's clinical conditions, which could affect appropriate maintenance of attention levels. Furthermore, it is recommended, if possible, to deepen the evaluation via tests that qualify as accurately as possible the patient's residual abilities and via the use of scales for the detection and quantification of potential affective impairments.

## Neuropsychological Rehabilitation

One of the targets of the neuropsychological assessment is to provide baseline data for the implementation of individualized rehabilitation programs, in which it is possible to draw up an intervention plan including cognitive exercises of increasing complexity and adequately calibrated to the difficulties that have arisen. A well-defined program examines and takes into account those factors that allow calibrating the level of commitment asked to the patient for an effective rehabilitation intervention that aims at recovering a deficit or maintaining current abilities over time. The cognitive sequelae of COVID-19 might also benefit from a specific and calibrated intervention on the symptoms profile of each patient. Even in this case, a vis-a-vis intervention allows controlling for relational variables that might be missed during online briefings. Yet, in the field of neuropsychological rehabilitation, it is certainly possible to think at activities that can be provided via teletherapy interventions without losing their effectiveness (5). Today, different tools are available to psychologists for the implementation of rehabilitation projects appropriate to the circumstances: such tools allow, for example, to remotely deliver the exercises, which are modulated in gradients of difficulty so that they can be adjusted to the actual level of impairment, and whose results can be monitored remotely by the psychologist with neuropsychological expertise.

## The Role of the Neuropsychologist

The number of patients affected by moderate and severe forms of COVID-19 raises serious considerations about the actual and forthcoming role of the psychologist with neuropsychological expertise. Based on the above, we believe that the contribution of the neuropsychologist should be considered in the phases that follow COVID-19 infection: in the course of neuropsychological intervention, which should be carried out as soon as possible in post-acute departments or rehabilitation facilities; in the implementation of cognitive treatment and behavioral deficits management, not only during the inpatient rehabilitation program but ensuring continuity of these interventions also after the discharge in outpatient facilities or via tele-monitoring; last but not least, the neuropsychologist must ensure the correct dissemination of information about the existence, the extent, and the consequences of patients' cognitive deficits to their caregivers and should provide them with adequate support also to cope with alterations of the emotional states.

## Some Conclusive Remarks

As scientific society of psychologists with neuropsychological expertise, we believe it is appropriate to point out the need for neuropsychologists' intervention in taking care of COVID-19 patients, considering that this pandemic, in its manifestation as neuro-COVID, may result in a variation of the epidemiological data on the incidence of cognitive alterations, in addition to the peculiarity of cognitive and behavioral alterations that follow acute necrotizing encephalopathy.

Also, all structures in which COVID-19 patients are hospitalized should be provided with information on cognitive, affective, and behavioral alterations resulting from this pathology.

Neuropsychological assessment of those patients should, then, be fostered, with the aim to correctly understand the deficit, implementing rehabilitation programs, managing cognitive problems in the family environment, and providing support for emotional difficulties.

We also believe that it is necessary to build a “network” of neuropsychologists in order to develop intervention protocols and collect data in the context of observational studies. Such research action could help define a better framework to understand and explain the cognitive consequences of the pathology, as well as enhance virtuous circles of cooperation and increase the homogeneity across neuropsychological interventions, with a view to drafting appropriate guidelines.

It should be finally underlined that the neuropsychologist, as psychologist, operates in close collaboration with colleagues dealing with other aspects of the human mind, aiming at an integrated and complete psychological care of the patient, her/his family system, and the social environment.

## Author Contributions

All authors listed have made a substantial, direct and intellectual contribution to the work, and approved it for publication.

## Conflict of Interest

The authors declare that the research was conducted in the absence of any commercial or financial relationships that could be construed as a potential conflict of interest.
